# Effect of Caffeic Acid and a Static Magnetic Field on Human Fibroblasts at the Molecular Level – Next-generation Sequencing Analysis

**DOI:** 10.1007/s12010-024-05094-z

**Published:** 2024-11-25

**Authors:** Magdalena Kimsa-Dudek, Celina Kruszniewska-Rajs, Agata Krawczyk, Anna Grzegorczyk, Agnieszka Synowiec-Wojtarowicz, Joanna Gola

**Affiliations:** 1 Department of Nutrigenomics and Bromatology, Faculty of Pharmaceutical Sciences in Sosnowiec, Medical University of Silesia in Katowice, Jednosci 8, Sosnowiec, 41-200 Poland; 2 Department of Molecular Biology, Faculty of Pharmaceutical Sciences in Sosnowiec, Medical University of Silesia in Katowice, Jednosci 8, Sosnowiec, 41-200 Poland

**Keywords:** Next-generation sequencing, Fibroblasts, Caffeic acid, Static magnetic field, Apoptosis

## Abstract

Due to their properties, numerous polyphenols and a static magnetic field could have therapeutic potential. Therefore, the aim of our research was to investigate the effect of caffeic acid (CA), a moderate-strength static magnetic field (SMF) and their simultaneous action on human fibroblasts in order to determine the molecular pathways they affect, which might contribute to their potential use in therapeutic strategies. The research was conducted using normal human dermal fibroblasts (NHDF cells) that had been treated with caffeic acid at a concentration of 1 mmol/L and then exposed to a moderate-strength static magnetic field. The RNA that had been extracted from the collected cells was used as a template for next-generation sequencing (NGS) and an RT-qPCR reaction. We identified a total of 1,006 differentially expressed genes between CA-treated and control cells. Exposure of cells to a SMF altered the expression of only 99 genes. Simultaneous exposure to both factors affected the expression of 953 genes. It has also been shown that these genes mainly participate in cellular processes, including apoptosis. The highest fold change value were observed for *HSPA6* and *HSPA7* genes. In conclusion, the results of our research enabled the modulators, primarily caffeic acid and to a lesser extent a static magnetic field, of the apoptosis signaling pathway in human fibroblasts to be identified and to propose a mechanism of their action, which might be useful in the development of new preventive and/or therapeutic strategies. However, more research using other cell lines is needed including cancer cells.

## Introduction

Polyphenols are the secondary metabolites of plants and fungi. Based on their carbon skeleton structure, they are grouped into phenolic acids, flavonoids, stilbenes and lignans. In plants, they not only play an important role in growth and development, but also in defending against stress. Moreover, due to their biological activity and properties, it is also possible to use them in medicine, pharmacology or cosmetology [1]. The experimental evidence suggests that natural plant compounds play a significant role in disease therapy, including cardiovascular, neurodegenerative diseases or cancer as well as in chemoprevention [2]. On the one hand, this is associated with their anti-inflammatory and antioxidant properties – the ability to scavenge free radicals, inhibit the production of reactive oxygen species (ROS) or activate the Nrf2 (NF-E2-related factor 2) pathway, which results in an increase in the antioxidant response of cells [3]. On the other hand, the presence of metal ions and higher concentrations of polyphenols promote their pro-oxidative properties, which in turn can promote cell apoptosis [4, 5]. This is important for supportive cancer therapy [6]. In addition, the adjunctive use of natural compounds might reduce the side effects of chemotherapy.

Caffeic acid appears to be a polyphenol that has a high therapeutic potential. It is a representative of the hydroxycinnamic acids, which comprise a subgroup of phenolic acids. Food sources of caffeic acid include legumes, tomatoes, nuts, cereals, sunflower seeds and various fruits. Because it is consumed with plant foods primarily in a bound form, absorption in the small intestine is crucial for its bioavailability. The availability of the bound form of hydroxycinnamic acids is dependent on hydrolysis and involves the intestinal microflora enzymes, ferulic acid esterases, after which caffeic acid can enter the cells *via* the monocarboxylic acid transporters (MCT) [7]. It positively impacts human health through its influence on the cell membranes, various signaling pathways and the cellular processes, which make its potential use in treating diseases such as cancer, diabetes, obesity, athlerosclerosis and neurodegenerative diseases possible [8–10].

The rationale for this research is the search for new therapeutic strategies supporting the treatment of many diseases, including cancer or diseases that are connected to free radical damages, the treatment of which is currently not fully effective. Therefore, more innovative methods are still being sought. Natural resources provide potential compounds with applications in clinical settings. Recently, it has been suggested that a combination of various factors might result in better therapeutic effects. Data on this subject are still fragmentary, especially with regard to the simultaneous use of both a chemical and physical agent. Significant scientific interest is focused on using a static magnetic field. It has been postulated that it can be used in the treatment of chronic pain [11], diabetes complications [12] and various cancers [13–16]. Recently, the possibility of using magnetic fields in drug delivery has also been postulated with promising results [17]. Moreover, there are indications of its possible synergistic effect together with chemotherapeutics.

Due to their wide occurrence in the human body and the ease of culturing them, fibroblasts are one of the most commonly used cells in in vitro studies. They play various roles in diseases, and act as mediators of inflammation and cancer development. Hence, they are useful as research models for many conditions as well as in drug toxicity studies and screening [18].

Despite numerous studies, the molecular mechanisms of the action of polyphenols are as yet not fully understood. The same problem is encountered with an SMF because its biological effects on cells are still controversial. Understanding the pathways of the action of various factors in the design of new therapeutic strategies is extremely important. This is enabled by using the modern screening techniques of molecular biology such as microarrays or next-generation sequencing [19]. To date, there have been only a few studies that have shown the effect of the simultaneous action of a chemical and physical agent on cells at the molecular level [13]. A detailed molecular analysis of their effect on cells will enable them to be used as a supportive therapy. Conducting screening studies using advanced molecular methods will enable the interesting pathways to be selected, which can later be transferred to detailed research in the design of new therapeutic strategies. Therefore, the aim of our research was to investigate the effect of caffeic acid, a moderate-strength static magnetic field and their simultaneous action on human fibroblasts in order to determine the molecular pathways they affect, which might contribute to their potential use in therapeutic strategies.

## Materials and Methods

The research was conducted using the normal human dermal fibroblast (NHDF cell line, Clonetics, CC-2511, San Diego, CA, USA) that had been treated with caffeic acid and simultaneously exposed to a moderate strength static magnetic field.

### Cell Culture Conditions

The NHDF cells were cultured in an FBM medium (Fibroblast Basal Medium, Lonza, Basel, Switzerland) with human fibroblast growth factor-basic (hFGF-B), insulin and gentamicin (FGM™ SingleQuots™; Lonza, Basel, Switzerland) at 37ºC in a 5% CO_2_ incubator (Heraeus, Hanau, Germany). The number and viability of the cells were assessed in a Countess™ Automated Cell Counter (Invitrogen, Carlsbad, CA, USA) after being stained with 0.4% trypan blue staining.

Cells between four and six passages were used for the experiments. The NHDF cells were seeded in 25 cm^2^ cell culture flasks at a density of 1 × 10^6^ cells (Sarstedt, Nümbrecht, Germany). After a 24 h incubation, the cells were treated with caffeic acid (CA) at a concentration of 1 mmol/L and then exposed to a moderate strength static magnetic field (SMF) with a magnetic induction of 0.7 T. Caffeic acid was purchased from Sigma-Aldrich, St. Louis, MO, USA (C0625, CAS No. 331-39-5). The stock solution of caffeic acid was prepared in phosphate-buffered saline (PBS, Lonza, Basel, Switzerland) and then diluted with the culture medium. The concentration of caffeic acid and the magnetic induction of the SMF were selected based on previous studies that had revealed the lack of their cytotoxicity to human cells [20, 21]. In this study, a static magnetic field was generated by permanent magnets in special patented tested chambers designed to study the influence of an SMF on cells in in vitro conditions. These chambers consisted of a ferromagnetic case and permanent magnets with a ferromagnetic yoke being the chamber bottom and cover. Permanent magnets were secured inside on the yoke and the chambers were closed with side, back and front walls. The internal dimensions of the chamber were adjusted to the dimensions of the culture flask. Non-magnetic distance plates determined the inner dimensions of the chambers, which were matched to the culture flask dimensions. The materials used for the chambers were as follows: N42SH magnets, *B*_*r*_ = 1.28–1.34 T, H_cB_ ≥ 955 kA/m, H_cJ_ ≥ 1512 kA/m, (BH)_max_ = 310–342 kJ/m^3^, S235JR steel and diamagnetic material. The chambers had a maximum operating temperature of 150 °C [22]. The design of the chambers enabled the magnetic field distribution in the central part of the chamber to be uniform, which was measured using a gauss meter. Unexposed cells were cultured in control chambers, where steel (0.0 T) was used instead of a permanent magnet.

The cells were grouped as follows: cells that had been treated with caffeic acid (chemical factor), cells that had been exposed to an SMF (physical factor), cells that had been simultaneosly exposed to CA and an SMF (mixed factor) and also the control non-treated cells. The cells that had been exposed to a chemical, physical, or both were incubated for 24 h. After this time, the cells were pelleted for further molecular analyses. The experiment was performed in duplicate.

### Molecular Analyses

The RNA was extracted from the collected cells and then the obtained nucleic acid isolates were used as a template for next-generation sequencing (NGS) and performing an RT-qPCR reaction.

#### Extraction of RNA

Total RNA was extracted using the TRIzol reagent (Invitrogen, Carlsbad, Calif, USA) according to the manufacturer’s instructions. Next, the RNA extracts were purified using an RNeasy Mini Kit (Qiagen Gmbh, Hilden, Germany) and DNase I (Fermentas International Inc., Ontario, Canada) according to the manufacturer’s protocol. The concentration of the extracts was assessed using the fluorometric method using a Quantus™ Fluorometer and the QuantiFluor^®^ RNA System (Promega, Madison, WI, USA). All of the samples were also analyzed using an Agilent 2100 Bioanalyzer and Agilent RNA 6000 Nano Kit (Agilent Technologies, Santa Clara, CA, USA) to evaluate the quality of the RNA and determine the RIN value (RNA integrity number).

#### Library Preparation and Sequencing

RNA extracts that met the qualitative and quantitative requirements were used for sequencing. The libraries were prepared using a TruSeq Stranded Total RNA Library Prep Kit and IDT for Illumina-TruSeq RNA UD Indexes (Illumina Inc., San Diego, CA, USA) according to the manufacturer’s protocol. Then, the quality of the libraries was determined using an Agilent DNA 1000 Kit and Agilent 2100 Bioanalyzer (Agilent Technologies, Santa Clara, CA, USA). Next, all of the samples were pooled, and this pooled library was sequenced using a NextSeq 500/550 High Output Kit v2.5 (150 cycles) and a NextSeq 550 (Illumina Inc., San Diego, CA, USA).

#### RT-qPCR Assay

The RT-qPCR technique was used to determine the mRNA level of the genes belonging to the heat shock protein family that were selected to validate the RNA-Seq analysis (*HSPA1A*, *HSPA1B*, *HSPA6*, *HSPA7*, *HSPA5* and *HSPB3*). The gene expression was evaluated using SYBR Green I chemistry (GoTaq^®^ 1-Step RT-qPCR System kit, Promega, Madison, WI, USA) and a LightCycler^®^ 480 Instrument II (Roche Life Science, Basel, Switzerland). The oligonucleotide primers were commercially available (Sigma-Aldrich, St. Louis, MO, USA).

A melting curve analysis was also performed to confirm the specificity of the amplification and the absence of any primer dimers. The mRNA copy number was determined based on a standard curve method that was previously described by Strzalka-Mrozik et al. [23].

### Statistical Analyses

The raw next-generation sequencing data quality was determined using a FastQC tool. Next, a Trimmomatic tool was used to filter out the poor quality reads and trim any poor quality bases from the samples [24]. Any low-quality nucleotides (base quality less than 20) were removed from each read at both ends. The reads were also scanned with a five nucleotide long window. The average quality in a window was required to be no less than 20. Only reads of at least 35 nucleotides in length were retained after the qualitative analysis after which the quality control was then re-performed using a FastQC tool. HISAT2 was used to map the next-generation sequencing reads (after building the index and indexing the human genome in the GRCh38 version). Converting to the BAM format, sorting and indexing the BAM files were performed using Samtools. The featureCounts function was used to calculate the number of mapped reads [25].

The EdgeR package was used to identify any differentially expressed genes (DEG) [26]. Genes with low expression were filtered based on their CPM values (counts per million) and a normalization of the library sizes. Subsequently, the samples that had been treated with the physical, chemical and both the physical and chemical agents were compared to the control samples to determine whether the gene expression level had changed significantly. Selected results are presented in diagrams that were created using the Glimma package [27].

A Gene Ontology and pathway enrichment analysis were performed using a PANTHER Classification System database. Additionally, sequencing analysis was conducted using an IGV tool (Integrative Genomics Viewer).

Statistical analyses of the RT-qPCR results were performed using Statistica 13.3 software (StatSoft, Tulsa, OK, USA). The data were analyzed by one-way ANOVA followed by Tukey’s post hoc tests. The values are expressed as the means ± SD and p values < 0.05 were considered significant.

## Results

**Fig. 1:**
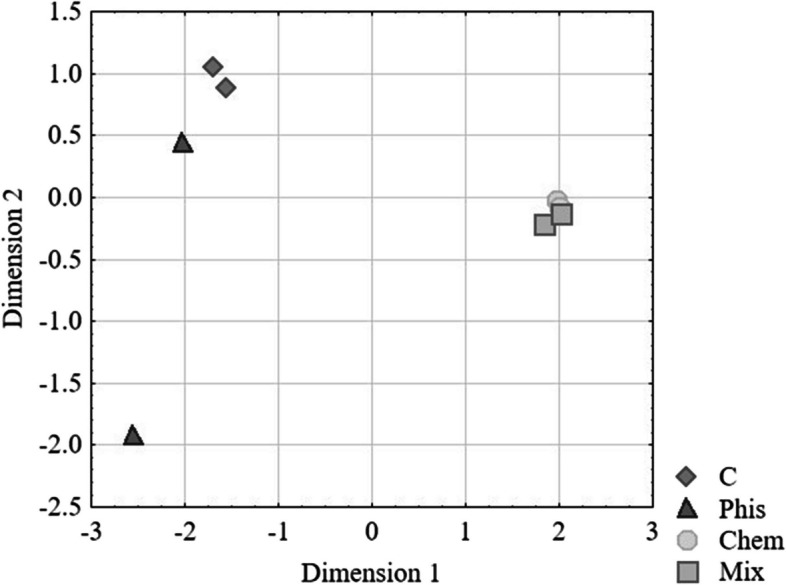
MDS plot that visualizes the level of the similarity of the transcriptome profiles of the control human fibroblasts (C) and the fibroblasts after exposure to a static magnetic field (Phis), caffeic acid (Chem) and both factors simultaneously (Mix)

A multidimensional scaling revealed that the cellular transcriptome profiles were different between the control cells (C) and the cells that had been exposed to an SMF (Phis) or that had been treataed with CA (Chem), whereas they were quite similar in the NHDF that had been treated with CA (Chem) and in the NHDF that had been simultanoeously exposed to a both factors (Mix) (Fig. 1).

### Differential Gene Expression Analysis

The first stage of the analysis was to identify the differentially expressed genes (DEGs) in the cells that had been treated with the chemical and physical factors. Samples that had been treated with caffeic acid at concentration of 1 mmol/L, samples that had been exposed to a moderate-strength static magnetic field (0.7 T) and samples that had been simultaneously exposed to a both factors were compared to the untreated control samples.

**Fig. 2:**
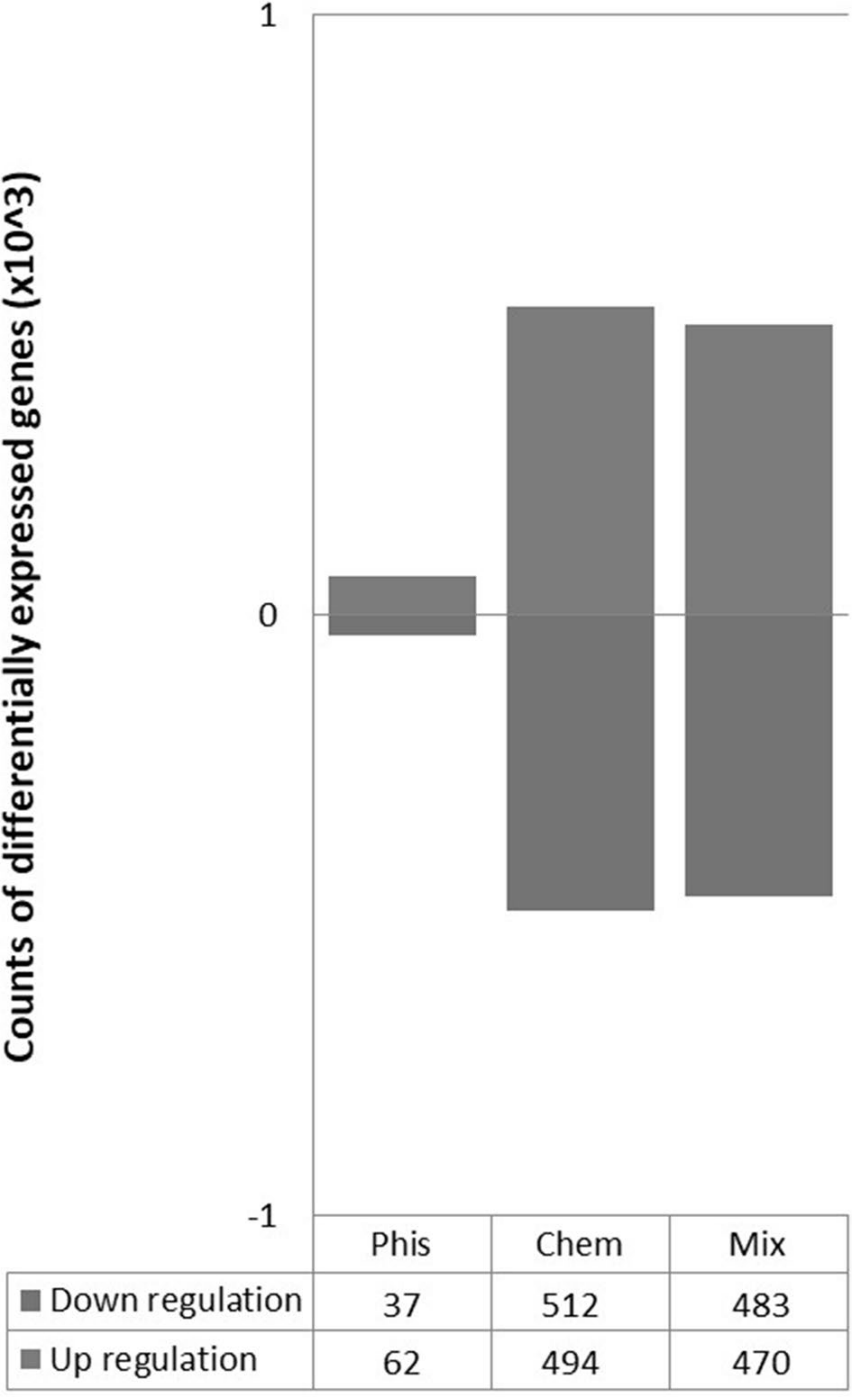
Counts of the differentially expressed genes in the human fibroblasts after exposure to a static magnetic field (Phis) and caffeic acid (Chem) and both factors simultaneously (Mix)

With the fold change FC ≥ 2.0 and *p* < 0.05 that was used, a change in expression of 1,006 genes for the CA-treated cells was observed. On the other hand, exposure of cells to a static magnetic field altered the expression of only 99 genes. Simultaneous exposure to both factors, chemical and physical, affected the expression of 953 genes. The obtained results suggest that at the molecular level human fibroblasts are mainly influenced by the tested polyphenol, not by a static magnetic field (Fig. 2).

**Fig. 3:**
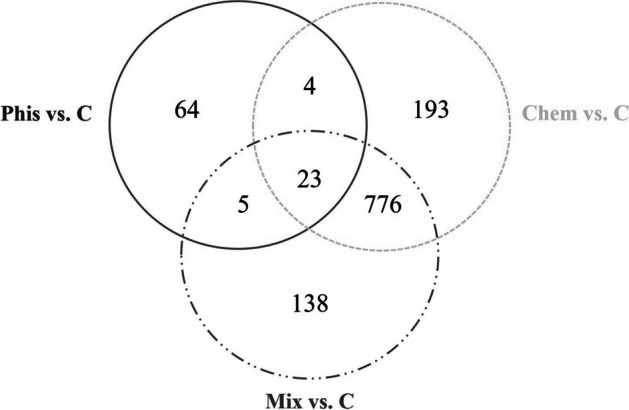
Counts of the common differentially expressed genes in the human fibroblasts after exposure to a static magnetic field (Phis) and caffeic acid (Chem) and both factors simultaneously (Mix)

The next stage of the analysis involved the selection of the differential genes that are common to the studied groups of cells. For this purpose, a Venn diagram was used (Fig. 3). This diagram showed the number of differentially expressed genes that are unique to cell treatment of physical factor (64 genes), chemical factor (193 genes) or both factors (138 genes) and those that are common for two (Phis and Chem – four genes, Phis and Mix – five genes, Chem and Mix – 776 genes) and three (23 genes) studied groups of cells.

### Gene Ontology and Pathway Enrichment Analysis

The next stage of the analysis included an assessment of which biological processes were influenced by the studied factors. The analysis was conducted for all of the common genes and separately for the genes that had changed only under the influence of CA, only under the influence of a SMF and under the influence of simultaneous action of both factors.

**Fig. 4:**
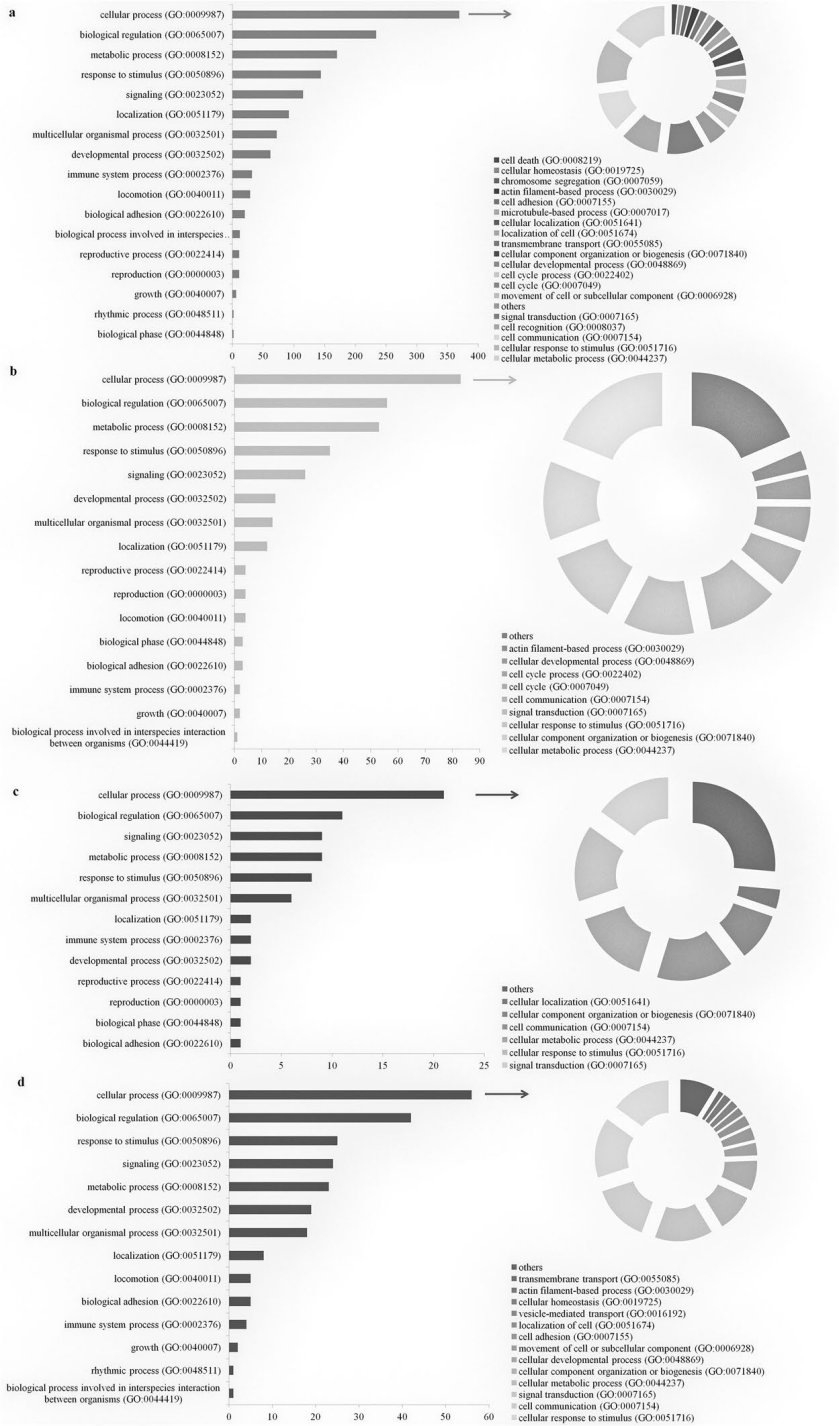
Subgroups of the common DEGs (**a**), the DEGs in the CA-treated cells (**b**), the DEGs in the SMF-exposed cells (**c**) and the DEGs in the cells that had been exposed to an SMF and simultaneously treated with CA (**d**) that are associated with the biological processes and the subcategories of the cellular process that were selected using the Panther 13.1 program

The PANTHER analysis showed that 17 biological processes were activated by the common differentially expressed genes, most of which are associated with the cellular process (GO:0009987). Next, this subcategory was explored in detail (Fig. 4a). To better understand the cellular processes that were influenced by a static magnetic field and caffeic acid, we further analyzed those genes that are associated with the cellular process in order to observe which pathways in which these genes participate are enriched. The pathway that was overrepresented was selected using the Bonferoni’s binominal test is apoptosis (Table 1).

**Table 1:** Overrepresented pathways with the genes that are associated with the cellular processes that were predicted using Bonferoni’s binominal test

PANTHER Pathways	Number of Genes observed/expected	Genes	Fold Enrichment	*p*
Apoptosis signaling pathway	10/2.01	*BCL2* – BCL2 Apoptosis Regulator	4.97	0.007
		*BCL2L10* – BCL2 like 10		
		*BCL2L11* – BCL2 Like 11		
		*CREM* – cAMP Responsive Element Modulator		
		*FOSB* – FosB Proto-Oncogene, AP-1 Transcription Factor Subunit		
		*HSPA1A* – Heat Shock Protein Family A (Hsp70) Member 1 A		
		*HSPA1B* – Heat Shock Protein Family A (Hsp70) Member 1B		
		*HSPA6* – Heat Shock Protein Family A (Hsp70) Member 6		
		*MAP2K3* – Mitogen-Activated Protein Kinase Kinase 3		
		*MCM5* – Minichromosome Maintenance Complex Component 5		

In the case of the DEGs, only those that were characteristic for the action of CA, for the action of an SMF and only for the simultaneous action of both factors, the largest number of genes also participated in the cellular processes (Fig. 4b-d). However, further analysis did not show that cellular process-related genes contributed to the pathways that were overrepresented.

These results suggest that both CA and an SMF primarily influence the cellular processes of normal human dermal fibroblasts, mainly apoptosis. It should also be noted that this effect is primarily caused by the bioactive compound polyphenol and under normal conditions, a static magnetic field alone slightly changed the gene expression.

### Validation of RNA-Seq Results by RT-qPCR Technique

Some common DEGs had significant differences in their expression between the studied groups of cells with the highest FC (FC ≥ 8), that is *HSPA6* and *HSPA7* genes. Additionally, the genes encoding the heat shock proteins (*HSPA1A*, *HSPA1B* and *HSPA6*) were associated with the overrepresented pathway. Hence, to validate the RNA-Seq results, we selected the genes encoding the heat shock proteins: *HSPA1A*, *HSPA1B*, *HSPA6* and *HSPA7*, as well as *HSPA5* and *HSPB3*, which were also changed under the influence of the examined factors (Table 2).

**Table 2:** Characteristics of the genes that were selected for validation using the RT-qPCR method

Gene Symbol	Gene Name	Phis vs. C	Chem vs. C	Mix vs. F
		logFC		
*HSPA1A*	*heat shock protein family A (Hsp70) member 1 A*	−0.265	3.794	3.561
*HSPA1B*	*heat shock protein family A (Hsp70) member 1B*	−0.268	3.851	3.598
*HSPA5*	*heat shock protein family A (Hsp70) member 5*	−0.798	1.785	2.413
*HSPA6*	*heat shock protein family A (Hsp70) member 5*	−1.472	8.248	7.989
*HSPA7*	*heat shock protein family A (Hsp70) member 7*	0.097	8.607	8.176
*HSPB3*	*heat shock protein family B (small) member 3*	−0.221	−1.977	−2.251

**Fig. 5:**
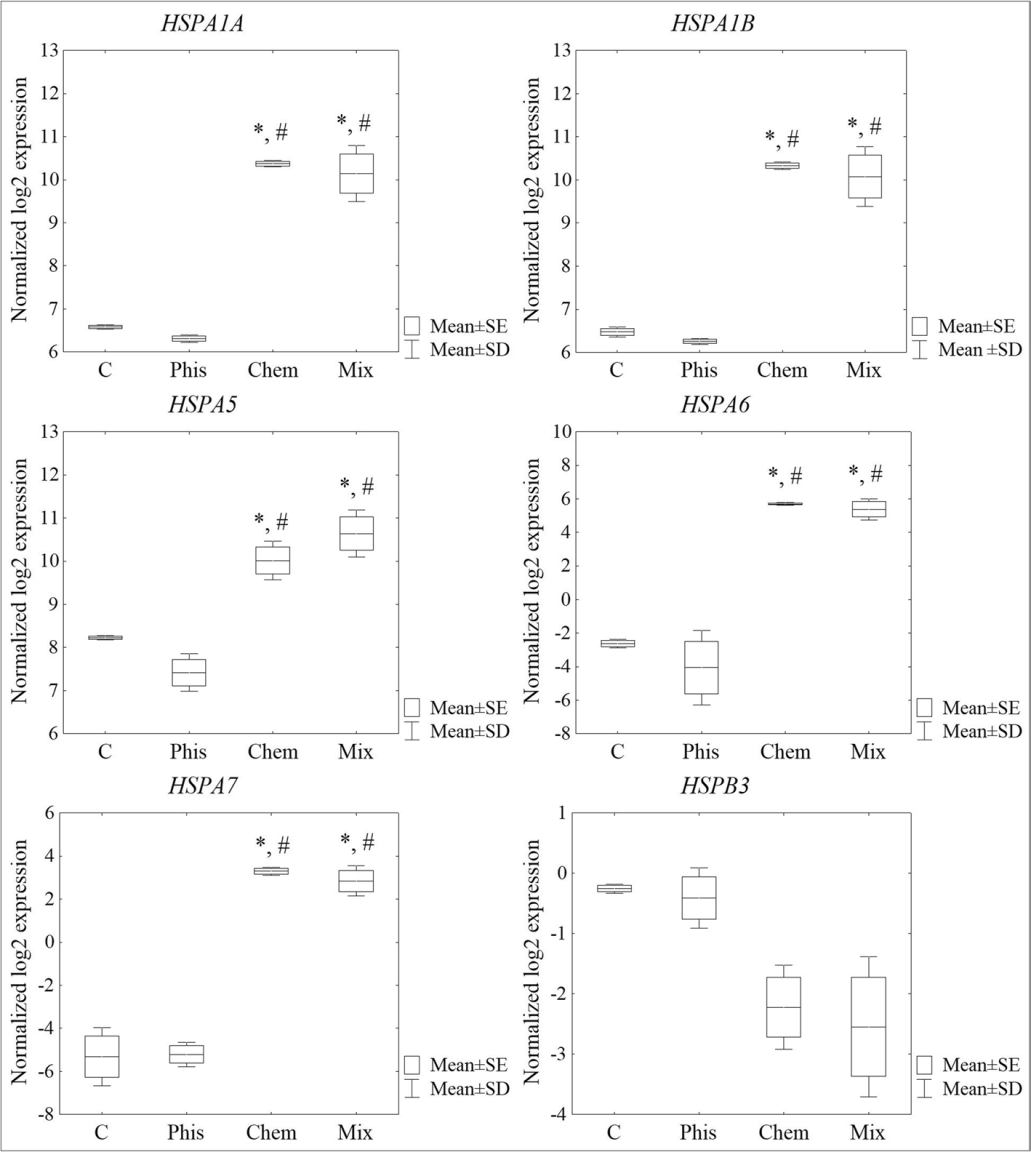
Expression of the genes encoding the heat shock proteins that were determined using RNA-Seq

**Fig. 6:**
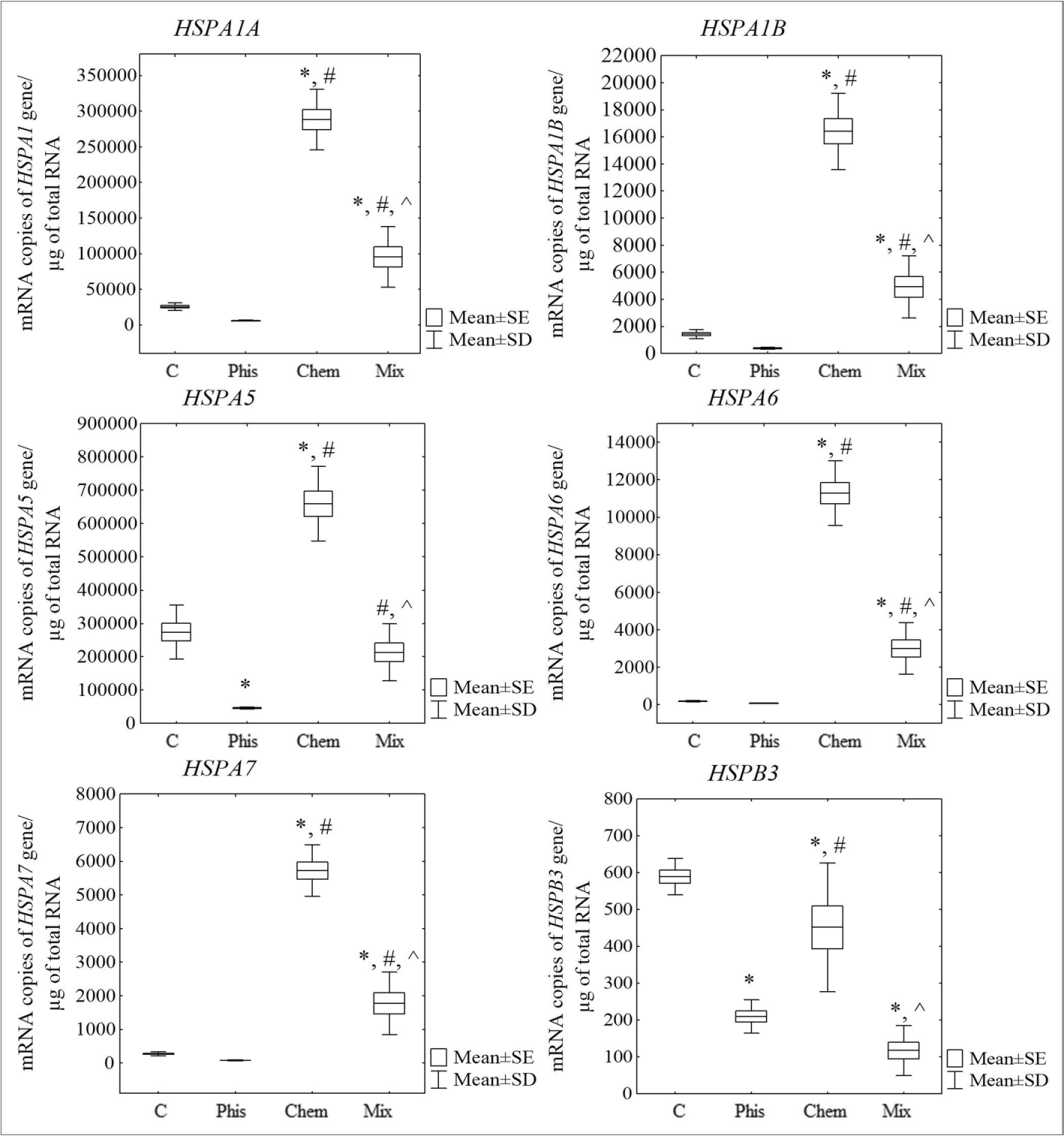
Expression of the genes encoding the heat shock proteins determined using the RT-qPCR technique

In the RNA-Seq results, it was observed that the genes encoding the heat shock proteins: *HSPA1A*, *HSPA1B*, *HSPA5*, *HSPA6* and *HSPA7* were significantly up-regulated in the cells that had been treated with caffeic acid (Chem) and simultaneously with both factors (Mix) (Tukey post hoc test, *p* < 0.05) compared to the control cells and the cells that had only been exposed to an SMF (Phis) (Fig. 5). There was no significant difference in the expression of *HSPB3* among the groups, but these genes were down-regulated with a tendency to statistical significance in the CA-treated cells and the cells that had been exposed to both factors compared to the control cells and the cells that had only been exposed to an SMF. The RT-qPCR method confirmed the expression results that were obtained using the NGS method, that is the overexpression of the *HSPA1A*, *HSPA1B*, *HSPA5*, *HSPA6* and *HSPA7* genes in the cells that had been treated with caffeic acid (Chem) and simultaneously with both factors (Mix) (Tukey post hoc test, *p* < 0.05) compared to the control cells and the cells that had only been exposed to an SMF (Phis) (Fig. 6). Moreover, in the case of *HSPB3*, there was a significant decrease in its expression in all of the studied groups of cells compared to the control (Tukey post hoc test, *p* < 0.05).

Moreover, a sequence analysis of the studied *HSP* genes did not indicate any changes under the influence of the examined factors.

## Discussion

Numerous studies on polyphenols have shown their potential for use in the treatment of many diseases as a supportive therapy. Similar properties have been observed in the case of a static magnetic field, the use of which in medicine has been widely discussed recently. Undertaking research to describe the effect of both factors on cells made it possible to explain the mechanism of their action, which in the long term will make it possible to propose new preventive and/or therapeutic strategies. Modern high-throughput screening techniques and bioinformatics tools allow the use of biologically active natural compounds and other factors in innovative drug design and development. Genomics, proteomics, metabolomics and their techniques play a special role [28, 29].

A number of studies have indicated that the effects of naturally occurring plant compounds as potential chemopreventive and/or therapeutic agents are their multiple signaling pathways, including Nrf2-ARE, NF-ĸB, p53 and apoptosis. Moreover, a static magnetic field, which affects the properties of the cell membranes and the concentration of cell secondary messengers, affects the signal transduction through various signaling pathways. However, as yet, it is not known whether the combined action of polyphenols and an SMF would have a synergistic effect, thereby enhancing their effects. Hence, it is necessary to understand key genes and their corresponding regulatory pathways that are involved in their mechanism of action. Therefore, in this study, RNA sequencing technology was used to analyze the differentially expressed genes in human fibroblasts that had been treated with caffeic acid, in cells that had been exposed to an SMF and in cells that had simultaneously been treated with both factors. Using RNA-Seq to reveal which genes and key pathways are affected by these agents could demonstrate the potential of both factors to treat diseases, including through molecularly targeted therapy, by knowing exactly where each agent works.

High-throughput sequencing is a modern method, which has many advantages over older technologies in gene expression studies such as microarrays, that is used in many biological/medical studies. One of these is its greater accuracy; the possibility to detect significant, slight changes in gene expression and the larger amount of information that can be obtained from a study. This sequencing method can also detect novel sequences, splice variants or non-coding RNA. Additionally, the small number of biological replicates in older gene expression techniques could result in experiments with a lower statistical power. For an RNA-seq analysis, reports indicate that two or three biological repeats be performed [30–32]. However, the costs of next-generation sequencing and complicated bioinformatics analysis can limit the use of this method [33]. Despite everything, RNA-seq is widely used, for example, in cancer research and new drug development [34, 35]. Hence, the decision to use this method in our study.

Similar research using Chem-seq and RNA-seq methods was performed by Atrahimovich et al. [36]. They indicated that other polyphenol – quercetin, influenced cellular function through change the expression of genes that are involved in the cell cycle, differentiation and development in human monocytes. They also suggested that these genes are related to the pro-apoptotic effect of quercetin on cancer cells. Moreover, the use of Chem-seq allowed them to examine direct binding sites of quercetin to the genome and may reflect its activity in the cell. This method allows for the study of the mechanism of influence of compounds on gene expression. The NGS method was also used in the study by Karousi et al. [37] to check the therapeutic potential of oleuropein and oleocanthal. These authors demonstrated the effect of these compounds from extra-virgin olive oil on gene expression in breast cancer cells, which participate in many processes, including cell death, apoptotic process, programmed cell death, response to stress, mitotic cell cycle process, cell division, and cancer progression. It initially suggests that this may be an alternative treatment option for this type of cancer. RNA-seq was also used to examine gene expression in the peripheral blood mononuclear cells (PBMC) of people after consuming apples enriched with polyphenols. It has been demonstrated that the consumption of such fruits may affect the functioning of the immune system [38]. In turn, other studies on fibroblasts treated with resveratrol imply the possibility of its use in the treatment of metabolic disorders through its effect on gene expression [39].

Differentially expressed genes that have been involved in apoptosis in SMF-exposed human umbilical cord mesenchymal stem cells was also revealed by RNA-sequencing in the study of Fang et al. [40]. NGS was also employed to identify DEGs in high static magnetic field condition in murine osteoclast precursor RAW264.7 cells in order to investigate the possibility of using magnetotherapy in bone diseases [41]. The above-mentioned and our research show the possibilities of using NGS technology in pharmacy and medicine in the drug design based on natural compounds and physical factors such as a SMF through identification of potential drug target genes.

Our studies have shown that apoptosis is an important pathway that is influenced by the action of caffeic acid and a static magnetic field. This process is essential for tissue homeostasis of the body and also is very significant as a therapeutic target of diseases including cancer [42]. Programmed cell death enables the elimination of damaged or unnecessary cells, which ensures cellular balance. It can also be induced by the pro-oxidant effect of antioxidants, which constitutes their potential in the treatment of diseases [43, 44]. The results from the PANTHER database indicated that among the genes that are involved in the overrepresented apoptosis signaling pathway, the *BCL2* gene encoding an anti-apoptotic factor was distinguished. In turn, the upregulation of the anti-apoptotic protein in cancer cells can promote tumor survival, which is an unfavorable phenomenon [45]. We have shown that both caffeic acid alone and together with a static magnetic field reduce its expression, which is important in the context of their possible use as potential therapeutic agents. Similar benefits may result from the overexpression of the *BCL2L11* regulator [46] under the influence of CA and a SMF as was demonstrated by RNA-seq analysis. Moreover, the effect of both studied factor on other genes encoding regulators of cell proliferation, such as *BCL2L10*, *MAP2K3* or *FOSB*, was also observed.

In addition to the apoptosis regulator mentioned above, the heat shock proteins (HSPs) play an important role in cell death processes [47]. Heat shock proteins are produced constitutively in cells, but their expression increases when cells are exposed to stress factors. In our study, it was observed that polyphenol along with the exposure to a static magnetic field caused the overexpression of the *HSPA1A*, *HSPA1B*, *HSPA5*, *HSPA6* and 7 genes belonging to the HSP70 family in the normal fibroblasts *versus* control cells. These proteins primarily play a cytoprotective role. Their cytoprotective effect in the case of normal cells is beneficial for the human body, while in the case of cancer, this action is undesirable and may contribute to the drug resistance of cancer cells. However, HSP70 inducers can be used to protect organs, e.g., when using chemotherapy [48]. It should also be remembered that HSP70 does not play an important role in carcinogenesis and tumor progression in every type of cancer as was shown by Ramp et al. [49] in their research on kidney cancer. In turn, silencing the *HSPB3* expression using chemical and physical factors seems to be beneficial for disease therapy. Kalioraki et al. [50] showed that its high expression is associated with poor relapse-free and overall survival in patients with colorectal adenocarcinoma.

Furthermore, the involvement of the HSP proteins in autophagy has been demonstrated, which, apart from apoptosis, is one of the essential pathophysiological mechanisms of modulating cell fate [51]. This can also be used in designing new therapeutic strategies in modern oncology [52].

This study has examined the impact of simultaneously exposition of human cells to caffeic acid and a static magnetic field using next generation sequencing technology. The findings clearly indicate that caffeic acid, also in combination with a static magnetic field, changes the expression of many genes, mainly related to biological processes, including apoptosis (such as *HSPA1A*, *HSPA1B*, *HSPA5*, *HSPA6*, *HSPA7* and *BCL2*). These findings have significant implications for the understanding of how these both factors act on human cells at molecular level. In general, it seems that caffeic acid in combination with a static magnetic field could be used as potential preventive or therapeutic agent. The most important limitation lies in the fact that the present research was performed on human normal cells. Therefore, further experimental investigations are needed to estimate the effect of chemical and physical factors also on different type of cells. Moreover, our results should be also confirmed at the protein level. In conclusion, the realization of our research enabled the modulators, primarily caffeic acid, and to a lesser extent a static magnetic field, of the apoptosis signaling pathway in human fibroblasts to be identified and to propose a mechanism of their action, which could be useful in the development of new preventive and/or therapeutic strategies. However, more research is needed using other cell lines including cancer cells.

## Data Availability

The data presented in this study are available upon request from the corresponding author.
